# Paravertebral Block Plus Thoracic Wall Block versus Paravertebral Block Alone for Analgesia of Modified Radical Mastectomy: A Retrospective Cohort Study

**DOI:** 10.1371/journal.pone.0166227

**Published:** 2016-11-09

**Authors:** Nai-Liang Li, Ben-Long Yu, Chen-Fang Hung

**Affiliations:** 1 Department of Anesthesiology, Koo Foundation Sun Yat-Sen Cancer Center, Taipei, Taiwan, Republic of China; 2 Department of Surgery, Koo Foundation Sun Yat-Sen Cancer Center, Taipei, Taiwan, Republic of China; 3 Biostatistics Section, Office of Clinical Research, Koo Foundation Sun Yat-Sen Cancer Center, Taipei, Taiwan, Republic of China; University of Bern, SWITZERLAND

## Abstract

**Background and Objectives:**

Paravertebral block placement was the main anesthetic technique for modified radical mastectomy in our hospital until February 2014, when its combination with blocks targeting the pectoral musculature was initiated. We compared the analgesic effects of paravertebral blocks with or without blocks targeting the pectoral musculature for modified radical mastectomy.

**Methods:**

We retrospectively collected data from a single surgeon and anesthesiologist from June 1, 2012, to May 31, 2015. Intraoperative sedatives and analgesic requirements, time to the first analgesic request, postoperative analgesic doses, patient satisfaction, and complications were compared.

**Results:**

Fifty-four patients received a paravertebral block alone (PECS 0), and 46 received a paravertebral block combined with blocks targeting the pectoral musculature (PECS 1). The highest intraoperative effect–site concentration of propofol was significantly lower in the PECS 1 group than in the PECS 0 group [2.3 (1.5, 2.8) vs 2.5 (1.5, 4) μg/mL, p = 0.0014]. The intraoperative rescue analgesic dose was significantly lower in the PECS 1 group [0 (0, 25) vs 0 (0, 75) mg of ketamine, p = 0.0384]. Furthermore, the PECS 1 group had a significantly longer time to the first analgesic request [636.5 (15, 720) vs 182.5 (14, 720) min, p = 0.0001]. After further adjustment for age, body mass index, American Society of Anesthesiologists Physical Status classification, chronic pain history, incidence of a superficial cervical plexus block placement, and operation duration, blocks targeting the pectoral musculature were determined to be the only significant factor (hazard ratio, 0.36; 95% confidence interval, 0.23–0.58; p < 0.0001). Very few patients used potent analgesics including morphine and ketorolac; the cumulative use of morphine or ketorolac was similar in the study groups. However, the incidence of all analgesic use, namely morphine, ketorolac, acetaminophen, and celecoxib, was significantly lower in the PECS 1 group [3.5 (0, 6) vs 5 (0, 12), p < 0.0001].

**Conclusions:**

Compared with the placement of a paravertebral block alone, combining blocks targeting the pectoral musculature with a paravertebral block for modified radical mastectomy reduced the sedative and analgesic requirements during operation and provided more effective postoperative analgesia.

## Introduction

Although paravertebral block (PVB) placement has been used as the sole anesthetic technique in breast cancer surgery [1,2], breast innervation [3,4] is complex, and a PVB cannot provide complete anesthesia for major breast surgery [4]. Epidural anesthesia has been used in breast cancer surgery to provide effective surgical anesthesia [5,6]. However, in case of bilateral sympathectomy and intercostal muscle blockade because of high thoracic epidural anesthesia reaching the T1–T5 levels, subsequent hypotension, bradycardia, and respiratory distress might occur [5–7].

Studies have reported that a PVB combined with general anesthesia [8] or sedatives [1,2] reduces pain and postoperative nausea and vomiting (PONV) after breast cancer surgery. A PVB combined with general anesthesia reduced the intraoperative propofol infusion rate and fentanyl requirement in breast cancer surgery [9]. However, major breast cancer surgery involves areas beyond the breast tissue, such as the pectoral musculature. A pectoral nerve block combined with a PVB was demonstrated to provide more effective pain management than did a PVB alone in patients undergoing reconstructive breast surgery [10]. However, the additional analgesic effects of a PVB combined with blocks targeting the pectoral region have not been studied in breast cancer surgery.

In this retrospective study, we compared a PVB with or without blocks targeting the pectoral region for modified radical mastectomy (MRM). We hypothesized that adding blocks targeting the pectoral region to a PVB provides more effective pain management in MRM. The primary aim of this study was to assess the intraoperative supplemental anesthetic effects of blocks targeting the pectoral region combined with a PVB. The secondary aims included determining postoperative analgesia and side effects of nerve blocks.

## Materials and Methods

The Institutional Review Board (IRB) of Koo Foundation Sun Yat-Sen Cancer Center reviewed and approved this retrospective study (KFSYSCC-IRB No.: 20150803A). The IRB exempted the requirement for informed consent. We retrospectively collected data from the postanesthesia registry and medical records. The study was conducted in accordance with the Declaration of Helsinki.

A PVB was used as the main anesthetic technique in major breast cancer surgery until February 2014, when we initiated combining it with blocks targeting the nerves innervating muscles of the pectoral region, namely the lateral pectoral nerve (LPN), medial pectoral nerve (MPN), long thoracic nerve (LTN), and thoracodorsal nerves (TDN), for patients undergoing MRM to obtain a more comfortable perioperative course. Eligible patients enrolled in the study were those who were diagnosed as having unilateral breast cancer and underwent MRM using a PVB with or without blocks targeting the pectoral musculature, supplemented with sedatives between June 1, 2012, and May 31, 2015. Patients who received a PVB alone between June 1, 2012, and February 2014 were included in the PECS 0 group. Moreover, patients who received a PVB combined with blocks targeting the pectoral musculature between February 2014 and May 31, 2015, were included in the PECS 1 group. The additional study inclusion criteria were as follows: the American Society of Anesthesiologists (ASA) Physical Status classification, I–III; age, 20–75 years; body mass index (BMI), <35 kg/m^2^; and the female sex. To ensure standardized operation and anesthetic techniques, we enrolled patients operated by a single surgeon (Ben Long Yu) and anesthesiologist (Nai Liang Li).

A departmental quality improvement measure involves conducting a routine follow-up of patients with a PVB for breast cancer surgery, with phone calls within 24 hours after discharge to exclude the late onset of pneumothorax or other untoward side effects. Nurse anesthetists telephone interviews with a standard format addressing complications and satisfaction to pain management; satisfaction was scored on a 4-point Likert scale (1 = poor, 2 = fair, 3 = good, and 4 = excellent). These data were entered into the postanesthesia registry. The principal investigator reviewed the medical records and postanesthesia registry and collected the following information: demographic and clinical characteristics, sedative and analgesic use during operation, time to the first request of postoperative analgesics, postoperative analgesic doses, patient satisfaction to pain management, and complications related to anesthetic techniques. On the completion of data retrieval, we performed deidentification by deleting patient ID after coding by the surgery date sequence, followed by data analysis.

The primary endpoints were intraoperative sedative and rescue analgesic requirements. The secondary endpoints were time to the first request of postoperative analgesics, postoperative analgesic requirement, and incidence of side effects.

### Procedure of Blocks

The patients were lightly sedated with intravenous midazolam (2.5–5 mg) and fentanyl (50–100 μg) before nerve block placement. We adopted the in-plane technique described by Renes [11] to place a PVB. Although a local anesthetic can longitudinally spread in the paravertebral space, a previous study [12] reported that the spread was not reliable after a single injection of a large volume of local anesthetics. Considering the comfort of patients and ability of a local anesthetic to spread to contiguous segments, we adopted multilevel injections at the T2, T4, and T6 levels.

Under ultrasound guidance, the wedge-shaped paravertebral space was located using a high-frequency probe (HFL38X linear probe, 6–13 MHz, SonoSite, Inc., WA, USA) placed lateral to the spinous process at the level of interest. A 22-gauge, 3.5-in spinal needle (Terumo^®^ spinal needle, Terumo Corporation, Tokyo, Japan) was subsequently inserted according to a previously described method [13]. Moreover, 8 mL of 0.5% levobupivacaine with 1:400,000 epinephrine was injected after negative aspiration of blood, air, or cerebrospinal fluid at the T2, T4, and T6 levels.

**Fig 1:**
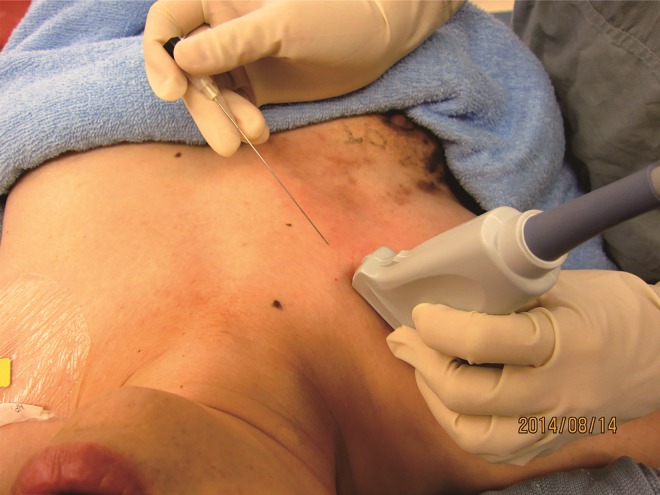
Probe orientation when performing blocks targeting lateral pectoral nerve.

**Fig 2:**
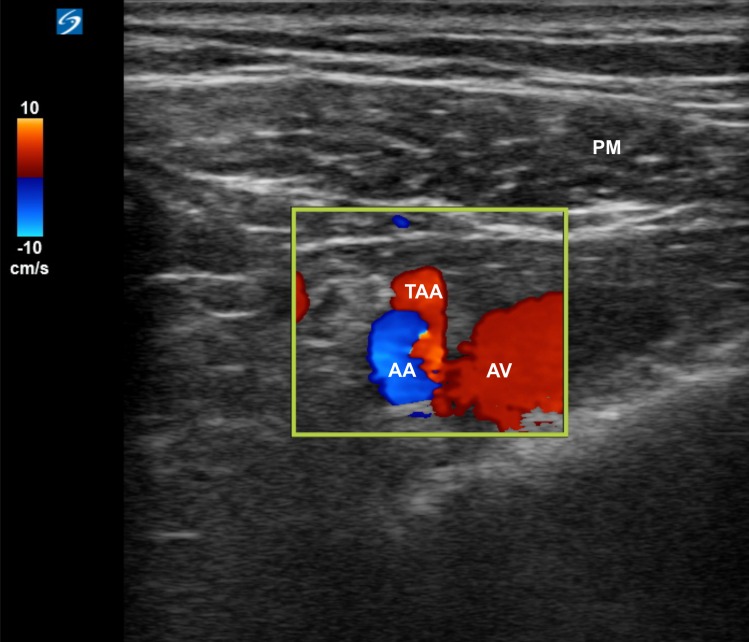
Ultrasound image of the thoracoacromial artery originating from the axillary artery. AA: axillary artery, AV: axillary vein, TAA: thoracoacromial artery, PM: pectoralis major muscle.

The patients were turned to the supine position after the completion of PVB placement. In the PECS 1 group, blocks targeting the pectoral region were placed following PVB placement. We placed the linear probe parallel to the clavicle and medial to the coracoid process (Fig 1) and visualized the pectoralis minor and major muscles, axillary artery, and vein. By pivoting the medial side of the probe, we identified the thoracoacromial artery at its origin from the axillary artery (Fig 2). Furthermore, with the needle in-plane with the probe from the medial to the lateral directions, we injected 3 mL of 0.25% levobupivacaine at the originating point of the thoracoacromial artery beneath the medial border of the pectoralis minor muscle after negative aspiration of blood. The needle was then withdrawn to the subcutaneous tissue. The probe was repositioned on the lateral third of the clavicle in a craniomedial to caudolateral orientation and was moved distally to identify the third rib. The needle was redirected in-plane with the probe from the previous puncture site. Moreover, 7 mL of 0.25% levobupivacaine was deposited between the lateral border of the pectoralis minor and serratus anterior at the third rib level.

**Fig 3:**
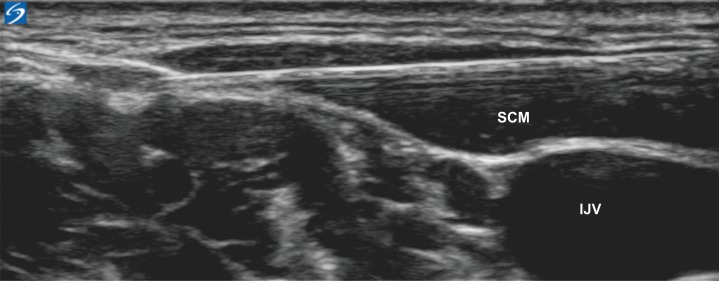
Ultrasound-guided superficial cervical plexus block. SCM: sternocleidomastoid muscle, IJV: internal jugular vein.

After the PVB and blocks targeting the pectoral region were placed, an ultrasound-guided superficial cervical plexus block (SCPB), as described by Herring et al [14], was placed for anesthetizing the upper part of the breast. In addition, 2 mL of 0.25% levobupivacaine was injected under the tapering posterolateral border of the sternocleidomastoid muscle just deep to the muscle belly but superficial to the deep fascia (Fig 3). If the total local anesthetic dose had reached the recommended dose limit, namely 3.0 mg/kg levobupivacaine, on completion of the placement of PVB and blocks targeting the chest wall, the SCPB was omitted. Supplemental intravenous analgesics were administered instead during dissection of the upper part of the breast.

### Sedation and Analgesia

Because of the departmental policy, the patients were not administered premedication before entering the operating room. They were administered midazolam (2.5–5 mg) and fentanyl (50–100 μg) immediately before the placement of the nerve blocks. After the efficacy of the blocks was confirmed by loss of sensitivity to cold on the area of interest, sedation commenced with the target-controlled infusion of propofol driven by the Schnider model in the effect–site control. The initial effect–site concentration (Ce) of propofol was approximately 1.2–1.5 μg/mL. We observed the response of the patients to names spoken in a normal tone. The propofol Ce dose was increased by 0.2–0.3 μg/mL every 3–5 minutes until the response of the patients to names spoken in the normal tone changed from lethargy [Observer Assessment of Alertness/Sedation (OAA/S) score [15], 4] to unresponsiveness (OAA/S score, 2–3). During the operation, incremental doses of propofol Ce (0.2–0.3 μg/mL) and/or the rescue analgesic ketamine (25 mg) were administered if the patients moaned or moved in response to surgical stimuli. These medications were administered at the discretion of the nurse anesthetists providing intraoperative care. Postoperative analgesics, namely morphine, ketorolac, celecoxib, and acetaminophen, were administered at the request of the patients. The analgesic choice was determined by the severity of pain and at the discretion of the anesthesiologists in the postanesthesia care unit and surgical staff in the ward. Morphine or ketorolac was administered if the patients experienced moderate to severe pain. Acetaminophen or celecoxib was administered if the patients experienced mild pain.

The following antiemetics were used during the operation: dexamethasone (4 mg), granisetron (1 mg), and droperidol (0.625 mg). A regimen of combining one or more antiemetics for emetic prophylaxis was followed according to each patient’s Apfel risk score and consensus guidelines [16,17].

### Statistical Methods

The null hypothesis of this study was the absence of differences between the two groups of patients undergoing MRM. The alternative hypothesis was that a PVB combined with blocks targeting the LPN, MPN, LTN, and TDN yields more effective pain management during and after MRM than does that without blocks targeting the aforementioned nerves.

We performed statistical analyses using SAS software (v 9.4; SAS Institute Inc., Cary, NC, USA). The chi-squared or Fisher exact test was used for comparing categorical variables. The Kolmogorov–Smirnov goodness-of-fit model was used to test the normality of continuous variables. Continuous variables that were not normally distributed were compared using the Wilcoxon two-sample test. Moreover, the median differences and their ranges were calculated. Continuous data with normal distribution were compared using the unpaired t test and are presented as the mean ± standard deviation. The time to the first request of analgesics was compared between the two groups by using the Kaplan–Meier estimate. Hazard ratios (HRs) of all clinical factors in the two groups were verified using the Cox proportional hazards model test. For all analyses, p < 0.05 was considered statistically significant.

## Results

The postanesthesia registry search yielded 108 patients who underwent unilateral MRM performed by Ben Long Yu and received nerve blocks and sedatives from Nai Liang Li from June 1, 2012, to May 31, 2015. All patients were women with an ASA classification of I–III. Two patients were excluded because they were aged >75 years on the surgery day; six patients who underwent MRM and tissue expander placement simultaneously and had been erroneously entered in the registry were also excluded. Of the remaining 100 patients, 54 and 46 constituted the PECS 0 and PECS 1, groups, respectively.

Demographic data of the two groups were similar. The data were comparable for chronic pain history, history of long-term analgesic use, risk of PONV, operation duration, and percentage of patients receiving the SCPB (Table 1).

**Table 1:** Patient demographics and clinical characteristics shown according to treatment arm.

Characteristics	PECS 0 (n = 54)	PECS 1 (n = 46)	p
Age (y)	51.4 ± 9.1	52.7 ± 9.7	0.49
BMI (kg/m2)	23.0 ± 3.0	23.1 ± 3.2	0.90
ASA status I/II/III, n/n/n	8/44/2	8/34/4	0.52
Chronic pain history, n (%)	2 (3.7)	4 (8.7)	0.29
Long term usage of analgesics, n (%)	0 (0)	0 (0)	-
Risk for PONV: low/medium/high, n/n/n	14/37/3	5/39/2	0.14
Duration of operation (min)	92.5 (56, 160)	97 (56, 140)	0.37
Combined SCPB, n (%)	33 (61.11)	20 (43.5)	0.08

Data are presented as n, n (%), or mean ± standard deviation. Duration of operation is given as median (min, max).

BMI: body mass index; PONV: postoperative nausea and vomiting; Risk of PONV: low risk denoted Apfel risk score of 1; medium risk denoted Apfel risk score of 2–3; high risk denoted Apfel risk score of 4; SCPB: superficial cervical plexus block; ASA status: American Society of Anesthesiologists physical status classification.

The highest intraoperative Ce of propofol was significantly lower in the PECS 1 group than in the PECS 0 group [2.3 (1.5, 2.8) vs 2.5 (1.5, 4) μg/mL, p = 0.0014]. The intraoperative rescue analgesic dose was significantly lower in the PECS 1 group [0 (0, 25) vs 0 (0, 75) mg of ketamine, p = 0.0384]. Time to the first request of analgesics was significantly longer in the PECS 1 group [636.5 (15, 720) vs 182.5 (14, 720) min, p = 0.0001]. Very few patients received potent analgesics, namely morphine or ketorolac; the number of patients using these two analgesics did not vary between the two groups. The cumulative dose of morphine or ketorolac was similar in both groups. Considering all analgesics (morphine, ketorolac, acetaminophen, and celecoxib), the incidence of analgesic use was significantly higher in the PECS 0 group. Patient satisfaction to pain management and hospital stay did not vary between the two groups (Table 2).

**Table 2:** Results.

Outcomes	PECS 0 (n = 54)	PECS 1 (n = 46)	p
Highest Ce (μg/mL)	2.5 (1.5, 4)	2.3 (1.5, 2.8)	0.0014
Intra-op Ketamine (mg)	0 (0, 75)	0 (0, 25)	0.0384
Time to the first request of analgesics (min)	182.5 (14, 720)	636.5 (15, 720)	<0.0001
Incidence of analgesic usage	5 (0, 12)	3.5 (0, 6)	<0.0001
Morphine used			0.23
yes	4	1	
no	50	45	
Ketorolac used			0.37
yes	7	9	
no	47	37	
Cumulative analgesic consumption			
Morphine (mg)	0 (0, 5)	0 (0, 3)	0.23
Ketorolac (mg)	0 (0, 90)	0 (0, 90)	0.36
Patient satisfaction			0.13
Score 1–3	7	2	
Score 4	47	44	
Hospital stay (hr)	45 (27, 96)	44 (24, 71)	0.37

Ce: effect-site concentration of propofol. Data are presented as n, or median (min, max)

**Fig 4:**
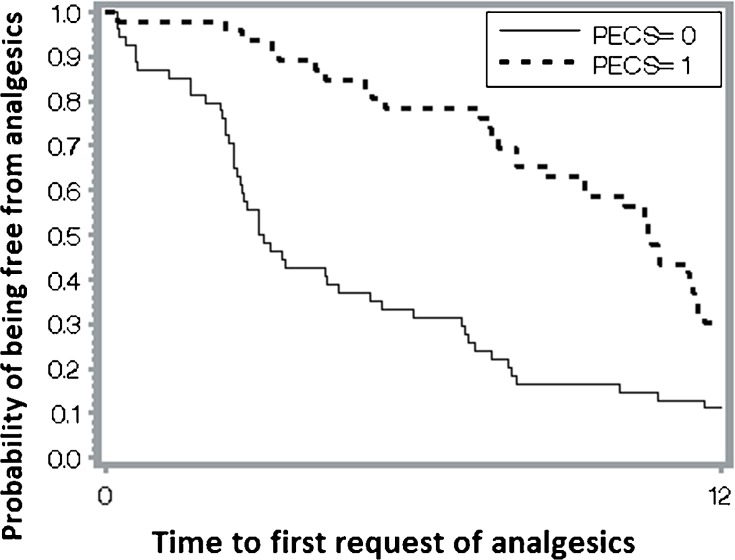
Time to the first request of analgesics. Kaplan Meier analysis illustrating the difference between the two groups in the proportion of patients with tolerable level of pain until the first request of analgesics. p = <0.0001, log-rank test.

Fig 4 presents the Kaplan–Meier plot for time to the first request of analgesics; the time was significantly shorter in the PECS 0 group (p < 0.0001). Cox regression analysis was used to identify crucial prognostic factors for time to the first request of analgesics. After adjustment for age, BMI, ASA, chronic pain history, operation duration, and combination SCPB, PECS 1 (vs PECS 0) was the only significant factor (HR, 0.36; 95% confidence interval, 0.23–0.58; p < 0.0001).

Very few patients experienced nerve block-related complications or PONV. Postoperative complications did not significantly vary between the PECS 0 and PECS 1 groups (Table 3).

**Table 3:** Complications.

Complications	PECS 0 (n = 54)	PECS 1 (n = 46)	p
**Nerve block-related**			
Venipuncture	1 (1.85)	0 (0)	0.54
Dysrhythmia	0 (0)	1 (2.2)	0.46
Injection site soreness	4 (7.4)	1 (2.2)	0.19
Injection site numbness	1 (1.85)	0 (0)	0.54
Hoarseness	1 (1.85)	0 (0)	0.54
**Sedation-related**			
Sore throat / dry mouth	4 (7.41)	2 (4.3)	0.08
PONV	3 (5.56)	1 (2.2)	0.15
Headache	1 (1.85)	1 (2.2)	0.50

PONV: postoperative nausea and vomiting. Data are presented as n (%).

## Discussion

Our data indicate that the combination of blocks targeting the pectoral girdle musculature and the PVB reduced sedative and analgesic requirements during MRM and provided more effective postoperative analgesia. Patients in the PECS 1 group had a longer duration of tolerable pain level before requesting analgesics, in addition to having a lower number of total analgesic doses.

A PVB does not encompass all nerve innervations of the chest. The upper part of the breast [4] and pectoral girdle musculature [18] are innervated by the cervical and brachial plexus, respectively. As early as 1951, a pioneer of regional anesthesia [19] recognized the necessity of integrating superficial cervical and brachial plexus blocks with intercostal nerve blocks for mastectomy.

In 2011, Guay et al [20] reported that an interscalene block with 20 mL of injectate could cover C3–C7. In his cadaver study, when an interscalene block was combined with a multilevel PVB (C8–T6), the minimum volume of a local anesthetic required to achieve complete anesthesia for breast surgery was 41 mL. Recent studies [21,22] have shown that the minimum effective volume of a local anesthetic required for ultrasound-guided interscalene block placement can be as low as 5 mL. Hence, the total volume of a local anesthetic to achieve complete anesthesia for breast surgery can be lower than that described by Guay et al. However, the brachial plexus block dose not only block the nerves of interest in breast surgery, namely the LPN, MPN, LTN, and TDN, but also blocks the nerves innervating the arm; therefore, we were concerned that postoperative numbness and weakness of the arm at the operated side and regional anesthesia may cause anxiety in patients, surgeons, and anesthesiologists. We adopted the technique described in a cadaver study [23] that selectively targeted the pectoral nerves with a minimal volume (3 mL) of a local anesthetic and avoided the spilling of the anesthetic to the nerves innervating the arm. One patient in the PECS 0 group experienced numbness at the injection site (upper back), which resolved within 5 days. No patient in either group experienced numbness or weakness of the arms when we adopted the approaches described in the present study.

Recently, Blanco et al [24] described block placement in breast surgery with axillary lymph node dissection, namely PECS II. Instead of a technique targeting specific nerves, PECS II is a thoracic wall fascial plane block using a relatively large volume of a local anesthetic to immerse the LPN, MPN, LTN, and lateral cutaneous branches of the T2–T4 intercostal nerves. The anterior cutaneous branches of the T2–T6 nerves, which supply the medial third of the breast, are spared. A Supplemental parasternal intercostal nerve block may be required for surgery involving this area. The upper part of the breast was not anesthetized by PECS II either. PECS II was previously demonstrated to have more favorable postoperative analgesia than did a single-level PVB for MRM [25]. Neither a single-level PVB nor PECS II alone provided complete anesthesia in radical breast surgery. In this study, regional anesthesia (PECS II or single-level PVB) was not used as the sole anesthetic technique but as an adjunct of general anesthesia maintained with isoflurane during MRM.

To reduce the dose of local anesthetics, we considered a combination of diluted levobupivacaine and low-dose opioid, because it has been reported to produce additive analgesic effects of each drug and reduce side effects for thoracotomy [26–28]. However, in a study on breast surgery, fentanyl plus diluted levobupivacaine improved analgesia but increased the PONV incidence [29].

We adopted a combination of blocks, namely a multilevel PVB, blocks targeting the pectoral girdle, and an SCPB. In our study, a minimum volume of 36 mL was required. The amount (150 mg of levobupivacaine) did not exceed the maximal recommended dose limit (3 mg/kg; S1 Table). The techniques and regimen we adopted were effective and safe. This regimen provided more effective pain management, including lower sedative and analgesic requirements and a longer tolerable pain duration, whereas no patient experienced local anesthetic toxicity, pneumothorax, or untoward weakness and numbness of the arms. The nerves innervating the pectoral musculature are typically considered pure motor nerves. The LPN and MPN from the brachial plexus have been suggested to carry a sensory component [18,30]. Aiyama (1973) [30] reported that through communications with the intercostobrachial nerve, the MPN may contain sensory fibers distributed to ventral surfaces of the arm, thoracic wall near the axilla, and inferolateral part of the pectoralis major. A few studies have reported pectoralis muscle spasm as a source of chest wall pain after tissue expander reconstruction [31] and radical mastectomy [32]. Studies have described a pectoral nerve block for use in patients undergoing subpectoral breast augmentation [10,23,33].

The LPN was demonstrated to run mostly (98%) along the pectoral branch of the thoracoacromial artery [34]. Moreover, 3 mL was adequate to block the LPN when approached at the originating point of the thoracoacromial artery [23]. Loukas et al [35] reported that the ansa pectoralis looped immediately distal to the thoracoacromial artery in 100% of specimens. In our study, we injected 3 mL of 0.25% levobupivacaine at the originating point of the thoracoacromial artery to block the proximal part of the LPN, the ansa pectoralis, which loops immediately distal to the thoracoacromial artery [18,35], and even the MPN nearby [18].

The local anesthetic we injected at the lateral border of the pectoralis minor and on the surface of the serratus anterior at the third rib level may have entered the axilla [24] and blocked the LTN [36] and TDN [37]. Theoretically, it may have also blocked the MPN because the MPN pierces the pectoralis minor at this level [18]. We arbitrarily selected a volume of 7 mL to maintain the total amount of local anesthetic within the maximal recommended dose limit. Additional studies are required to determine the minimum effective volume. Guay et al [20] validated that 2 mL was adequate to completely block the superficial cervical plexus.

Although the results are encouraging, the feasibility of this practice would be challenged if the efficiency of operating room turnover is assigned high priority in the absence of separate block rooms or a well-equipped (ordinary vital sign monitors and an ultrasound machine) preoperative holding area. The placement of the additional blocks required extra time. Typically, 10 minutes was adequate for placing the multilevel PVB; however, we spent additional 15 minutes to place the blocks targeting the pectoral musculature and the SCPB.

Our study had some limitations. First, to minimize the bias produced by different levels of proficiency or inconsistent practice among surgeons and anesthesiologists, we enrolled patients operated by a single surgeon and anesthesiologist. However, we retrospectively compared data between the two historical groups. The study duration was more than 3 years. The maturation of the skills of the surgeon and anesthesiologist over this period could have introduced a proficiency bias in this study, yielding more favorable PECS 1 results. The retrospective study design without masking the investigator was another limitation. The anesthesiologist’s awareness of the anesthetic technique may have introduced an expectancy bias, influencing the propofol Ce in the PECS 1 group. Third, this study included a small sample size. Therefore, it was difficult to determine significant differences in certain outcomes with clinical effects such as the length of hospital stay and opioid consumption. Although we observed that combining blocks targeting the pectoral musculature with a PVB improved pain management for MRM, the causal relationship conclusion must be definitively established by future double-blinded randomized studies. Fourth, the optimal volume of a local anesthetic required in the pectoral region must be investigated. Future volunteer or cadaver studies for local anesthetic spread are required to further contribute to the body of knowledge on this topic.

## Conclusion

Combining a multilevel PVB with blocks targeting the pectoral musculature reduced intraoperative sedative and analgesic requirements, increased the duration to the first analgesic request, and reduced postoperative analgesic doses, compared with the use a PVB alone for MRM.

## Supporting Information

S1 Data The clinical data set. (XLS)

S1 File English editing certificate. (PDF)

S1 Table Regimen of blocks for the PECS 1 group. (DOC)
