# Supplementary material for: Paravertebral Block Plus Thoracic Wall Block versus Paravertebral Block Alone for Analgesia of Modified Radical Mastectomy: A Retrospective Cohort Study
Source: PLoS One. 2016 Nov 9;11(11):e0166227. doi: 10.1371/journal.pone.0166227 (PMC5102399; doi:10.1371/journal.pone.0166227)
Supplement: S1 File — (PDF) [file pone.0166227.s002.pdf]

Wallace Academic Editing

English Editing Certificate

This certifies that the paper **Paravertebral Block Plus Thoracic Wall Block Versus Paravertebral Block Alone for Analgesia of Modified Radical Mastectomy: A Retrospective Cohort Study** has been edited by Amy Adams on September 18, 2016 and is considered to be improved in grammar, punctuation, spelling, verb usage, sentence structure, conciseness, general readability, writing style, and native English usage to the best of the editor's ability.

Amy Adams

Best regards,
Wallace Academic Editing

Phone No.: +886-2-2555-5830
Website: http://www.editing.tw
Email: editing@wallace.tw
Address: 3F., No.180, Chang'an W. Rd., Datong Dist., Taipei City
