# Supplementary material for: Paravertebral Block Plus Thoracic Wall Block versus Paravertebral Block Alone for Analgesia of Modified Radical Mastectomy: A Retrospective Cohort Study
Source: PLoS One. 2016 Nov 9;11(11):e0166227. doi: 10.1371/journal.pone.0166227 (PMC5102399; doi:10.1371/journal.pone.0166227)
Supplement: S1 Table — (DOC) [file pone.0166227.s003.doc]

**S1 Table. Regimen of blocks for the PECS 1 group (multilevel PVB, blocks for pectoral girdle, and SCPB) in our study.**

| Block | Target (volume) | Concentration of levobupivacaine | Dose |
| --- | --- | --- | --- |
| PVB | T2-4-6 (8 ml - 8 ml - 8 ml) | 0.5 % | 120 mg |
| Pectoral | LPN + AP ± MPN (3 ml)  LTN ± TDN ± MPN (7 ml) | 0.25 %  0.25 % | 25mg |
| SCPB | SCP (2 ml) | 0.25 % | 5mg |

PVB: paravertebral block, LPN: lateral pectoral nerve, MPN: medial pectoral nerve, AP: ansa pectoralis, LTN: long thoracic nerve, TDN: thoracodorsal nerve, SCP: superficial cervical plexus, SCPB: superficial cervical plexus block.
